# Dietary patterns and metabolic syndrome amongst adult residents: A cross-sectional study in a rapidly urbanized Southern Chinese city

**DOI:** 10.1097/MD.0000000000039692

**Published:** 2024-09-27

**Authors:** Maozhen Fu, Dandan Yang, Yan Luo, Yuliang Zou

**Affiliations:** a Shenzhen Pingshan District Center for Disease Control and Prevention, Shenzhen, China; b Department of Global Health, Center of Health Management, School of Public Health, Wuhan University, Wuhan, China.

**Keywords:** cross-sectional study, dietary patterns, factor analysis, metabolic syndrome, urbanization

## Abstract

We aimed to investigate and summarize dietary patterns and explore the association between dietary patterns and metabolic syndrome (MS) and its components among adult residents in a rapidly urbanized city. We employed a multi-stage random sampling method to select 1000 adult residents who underwent a comprehensive survey, including questionnaires, physical examinations, and laboratory tests. The diagnosis of metabolic syndrome was made when the participant met 3 or more of the 5 criteria outlined in the “2017 Chinese Guidelines for the Prevention and Treatment of Type 2 diabetes.” Factor analysis and a nonconditioned logistic regression model were used. Nine hundred seventy-five participants with a mean (SD) age of 41.08 (11.06) were included. The prevalence of metabolic syndrome was 19.4% (n = 189). Significant differences were observed between the MS and non-MS groups in terms of patient characteristics in terms of sex (*P* < .001), age (*P* < .001), education (*P* < .001), marital status (*P* = .025), smoking (*P* < .001), and alcohol consumption (*P* = .044). Three dietary patterns were summarized: traditional, coastal, and meat. The coastal pattern was associated with a significantly lower prevalence of MS (*P* < .001), elevated blood pressure (*P* < .001), and high triglyceride levels (*P* = .03). However, in the multivariate analysis, we found no significant associations between dietary patterns and MS or its components after adjusting the demographic characteristics and behaviors, even when the *P*-value was close to .05. In this study, we did not find an association between dietary patterns and MS and its components after adjusting covariates as much as possible in Pingshan, Shenzhen, a rapidly urbanized city, but underscore the potential health benefits of the coastal dietary pattern, which highlights the importance of conducting further research for a comprehensive understanding.

## 1. Introduction

Metabolic syndrome (MS) is a multifaceted clinical syndrome characterized by central obesity, insulin resistance, elevated blood pressure, and dyslipidemia.^[1]^ The prevalence of this syndrome is a substantial concern at global and national levels, posing a significant public health challenge. In the United States, the prevalence of MS among adults increased from 37.6% in 2011 to 41.8% in 2018.^[2]^ Interestingly, as a developed East Asian nation, South Korea prevalence of MS differs from that of the United States. A study conducted in South Korea reported a 26.1% prevalence of MS among Korean adults from 2004 to 2013.^[3]^ Similarly, between 2015 and 2017, the standardized prevalence of MS in Chinese adults aged 20 years and above was 31.3%.^[4]^ Even when employing diverse diagnostic criteria for MS, the high prevalence among the Chinese population persists.^[5]^ Notably, individuals affected by MS who do not receive timely intervention and treatment face an elevated risk of developing diabetes and cardiovascular diseases,^[6]^ thereby contributing to the overall disease burden.

Building on prior epidemiological research, multiple studies conducted in recent decades have investigated the factors contributing to the onset of MS. The significant increase in the prevalence of MS can be largely attributed to shifts in lifestyle, socioeconomic status, and dietary habits. Several studies have uncovered connections between dietary components and MS.^[7,8]^ Evaluating the isolated impact of individual nutrients or dietary elements has inherent limitations because of the intricate interactions between various food components and nutrients. Consequently, dietary patterns have emerged as a more robust method for predicting disease risk with greater accuracy than the examination of individual constituents. Dietary patterns provide a more holistic evaluation of diverse food components and nutrients, offering a realistic and precise representation of the overall dietary behavior within populations. A comprehensive meta-analysis of 85 observational studies suggested that adherence to a “healthy” dietary pattern, characterized by elevated consumption of vegetables, fruits, poultry, fish, and whole grains, may correlate with a reduced risk of MS development. By contrast, adopting a “meat/western” dietary pattern, predominantly characterized by the intake of red meat, processed meat, animal fats, eggs, and sugary foods, is linked to an elevated risk of MS.^[9]^

In China, regional disparities in geographical location, social culture, and economic conditions have contributed to distinctive dietary patterns that reflect local characteristics. Consequently, analyses of the association between diverse dietary patterns and MS have produced heterogeneous findings. A study conducted in Jiangsu Province identified 3 prevalent dietary patterns: a modern pattern characterized by pork, poultry, vegetables, seafood, and pastries; a plant oil/seasoning/soy product pattern centered on vegetable oil, seasonings, salt, and soy products; and a modern high-wheat pattern featuring wheat, tubers, fruits, and meat. The findings indicated a positive correlation between modern and modern high-wheat dietary patterns and MS risk, whereas the plant oil/seasoning/soy product pattern demonstrated a negative association.^[10]^ Conversely, a study in Sichuan Province outlined 4 dietary patterns: a coarse grain and soy pattern emphasizing high intake of coarse grains and soy; a meat and fruit pattern with elevated consumption of poultry, livestock meat, and fruits; a street snacking pattern involving snacks, fried foods, nuts, and beverages; and a minimalist satiety pattern based on refined grains with pickled vegetables, potatoes, and vegetables. Notably, no significant association was observed between dietary pattern and MS in this study.^[11]^ Discrepancies across regional studies underscore the importance of region-specific investigations into the relationship between dietary patterns and MS, while acknowledging local nuances.

This study was based on the 2018 Chronic Disease and Risk Factors Monitoring Project conducted in Pingshan, Shenzhen, China. The Pingshan District possesses unique urban characteristics, characterized by a high degree of population mobility, a youthful age structure, cultural diversity, and the aggregation of various industries. The city is undergoing rapid development, serving as a microcosm for China’s swift urbanization process. We selected the Pingshan District in Shenzhen as the research location with the aim of investigating and understanding the dietary habits of urban adults from diverse cultural backgrounds. Our study sought to explore the relationship between dietary patterns and MS in adults, thereby providing a scientific basis for the prevention and control of MS. Simultaneously, we aimed to offer dietary guidance and intervention strategies for individuals with MS.

## 2. Methods

### 2.1. Participants

**Figure 1.**
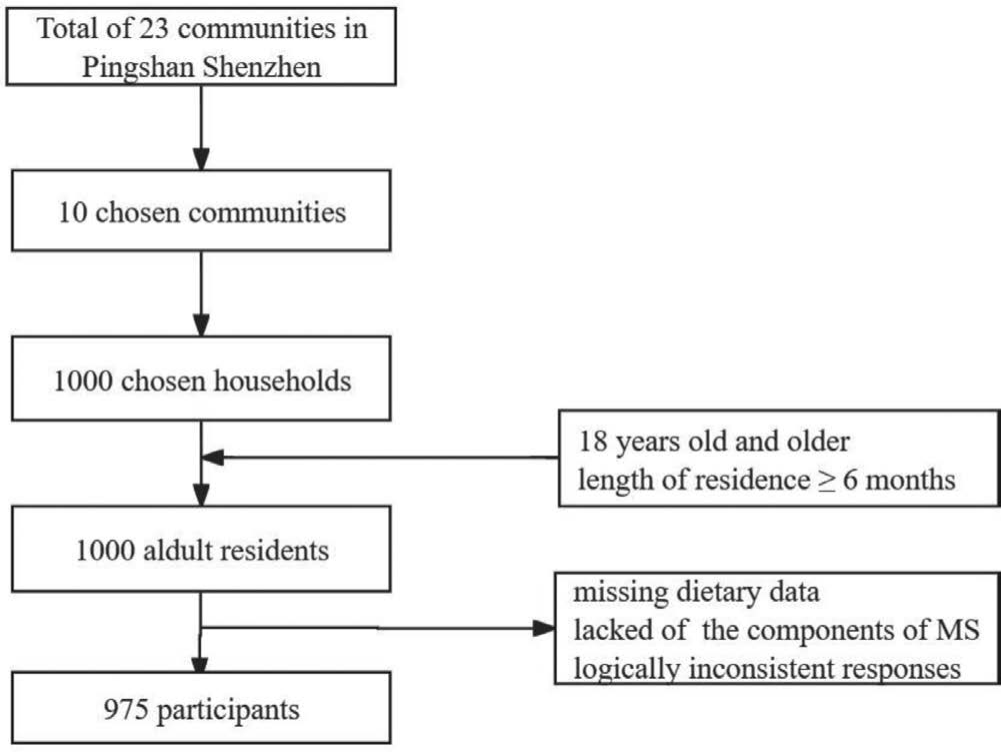
Flowchart of sample selection.

We adopted a multi-stage random sampling approach. Among the 23 communities in Pingshan District, a random selection procedure was used to designate 10 communities. Subsequently, 100 households were randomly designated within each selected community. Within each household under consideration, individuals aged 18 years and older, who held a permanent resident status, were selected through a random process utilizing the KISH table method. In the initial sampling phase, 1000 individuals were included. Subsequently, participants were refined by excluding those with missing dietary data, individuals exhibiting logically inconsistent responses, and those who lacked information pertaining to the MS components. This meticulous filtering process ultimately resulted in the inclusion of 975 participants in our study. The process of sample selection is shown in Figure 1.

### 2.2. Data collection

#### 2.2.1. Questionnaires

Sociodemographic characteristics, health-related behaviors, medical history, and dietary habits of the study participants were collected through structured questionnaires.

In our study, education was divided into 4 levels, namely Elementary school, Middle school, High school, and College degree or above. Occupation was divided into 3 types. Type I includes agricultural, forestry, animal husbandry, fishery and water conservancy production personnel, transportation equipment operators, commercial and service personnel, and other workers. Type II includes personnel from government agencies, party and mass organizations, enterprises, public institutions, professional and technical personnel, military personnel, and students. Type III includes unemployed, household workers, and retired personnel.

Dietary evaluation was conducted using a food frequency assessment that ascertained the frequency and mean consumption of 13 food items including soft and other sugary drinks over the previous year. This study focused more on the impact of fresh, minimally processed foods on metabolic syndrome, and therefore selected 9 types of minimally processed foods for investigation. The 9 types of foods included: grains, vegetables, fruits, poultry, red meat, freshwater fish, seafood, eggs, and dairy products, which was fresh and without special processing.

Smoking status was categorized as current smoker, former smoker, or nonsmoker. Alcohol intake within the past 12 months was defined as having alcohol consumption.

Physical activity levels were assessed using the calculation method of the International Physical Activity Questionnaire.^[12]^ This approach encompasses the classification of physical activities into 3 primary types: occupational, transportation, and leisure time, with each assigned intensity value. Subsequently, an aggregate physical activity score was computed and categorized into 3 groups: high, moderate, and low physical activity levels.

Sedentary behavior refers to physical activities undertaken while in a seated or reclining position with an energy expenditure of <1.5 metabolic equivalents during wakefulness. In the questionnaire survey, respondents were asked: “on a typical day, how much time do you spend sitting, reclining, or lying down? (Including time spent sitting while working, studying, reading, watching TV, using a computer, resting, and other sedentary behaviors, but excluding time spent sleeping)” to investigate the duration of sedentary behavior per day among the participants.

#### 2.2.2. Physical examinations and laboratory tests

Waist circumference, blood pressure, fasting blood glucose, total cholesterol, triglyceride (TG), and high-density lipoprotein cholesterol (HDL-C) levels were assessed using a combination of physical examinations and laboratory tests after fasting, which means not eating any food for at least 10 to 12 hours, except for drinking water. Waist circumference, is measured by trained researchers at the horizontal circumference of the navel center or the midpoint line between the lowest point of the rib and the upper edge of the iliac crest, using a standardized and accurate measuring tape, at the end of exhalation and before inhalation begins. Blood pressure measurement and blood collection are carried out by doctors and nurses from the local community health service canter. By the guidelines suggested by Chinese Clinical Practice Guidelines for Hypertension (2022 Edition), blood pressure was carefully recorded with the aid of standardized mercury sphygmomanometers, involving 3 consecutive measurements taken at one-minute intervals, with the mean value subsequently documented. Fasting blood glucose is measured by collecting venous blood samples and using the glucose oxidase–peroxidase coupled method for measurement. Enzyme method was used for serum total cholesterol and TG determination, while homogeneous method was used for serum HDL-C determination.

#### 2.2.3. Diagnostic criteria for MS

The diagnosis of MS was based on the criteria outlined in the “2017 Chinese Type 2 Diabetes Prevention and Treatment Guidelines”^[13]^ as follows: (1) abdominal obesity: a waist circumference of ≥ 90 cm for males and ≥85 cm for females; (2) high blood glucose: fasting blood glucose of ≥6.1 mmol/L, or two-hour post-load blood glucose ≥7.8 mmol/L and/or a confirmed diagnosis of diabetes under treatment; (3) elevated blood pressure: blood pressure of ≥130/85 mm Hg and/or a confirmed diagnosis of hypertension under treatment; (4) fasting TG of ≥1.70 mmol/L; (5) fasting HDL-C levels <1.04 mmol/L. MS was diagnosed when an individual satisfies 3 or more of the above criteria.

#### 2.2.4. Statistical analysis

Factor analysis was used to extract the dietary patterns, incorporating the intake of various food categories into the factor analysis model. Prior to the analysis, the Kaiser–Meyer–Olkin test and Bartlett sphericity test were conducted to assess whether the sample was suitable for factor analysis. The inclusion criterion for common factors was that eigenvalues should be >1. To better categorize the original variables, orthogonal varimax rotation was applied to the initial factors. The main dietary patterns were determined using scree plots, eigenvalues, rotated factor loadings, variance contributions, and nutritional knowledge.

Statistical analysis was performed using SPSS version 26.0. Quantitative data that followed or closely approximated a normal distribution were presented as (x̅ ± s), whereas count data were presented as [n (%)]. Rates were compared using the chi-squared (χ²) test, and the Bonferroni method was used for multiple comparisons between groups. Logistic regression analysis was used to explore the association between different dietary patterns and MS and its components. All statistical tests were two-tailed, and a significance level of *P* < .05 was considered statistically significant.

## 3. Results

### 3.1. Characteristics of participants

The study included 975 participants, with a prevalence of MS of 19.4% (n = 189). The participants had a mean (SD) age of 41.08 (11.06), with 465 (47.7%) males and 510 (52.3%) females. In terms of occupation, 488 (50.1%) participants were categorized as Type I, 222 (22.8%) as Type II, and 265 (27.2%) as Type III. The proportion of individuals with a sedentary behavior time equal to or >6 hours is the highest among the total population, accounting for 43.38% (n = 432). Statistical analysis revealed significant differences (*P* < .05) between the MS and non-MS groups in terms of sex, age, education, marital status, smoking status, and alcohol consumption (Table 1).

**Table 1:** Characteristics of participants.

Variable	Total (n = 975)	MS (n = 189)	Non-MS (n = 786)	χ^2^	*P*
Sex				50.61	<.001
Male	465 (47.7)	134 (70.9)	331 (42.1)		
Female	510 (52.3)	55 (29.1)	455 (57.9)		
Age				34.17	<.001
19–29	126 (12.9)	13 (6.9)	113 (14.4)		
30–39	379 (38.9)	55 (29.1)	324 (41.2)		
40–49	241 (24.7)	49 (25.9)	192 (24.4)		
50–59	157 (16.1)	51 (27.0)	106 (13.5)		
60–	72 (7.4)	21 (11.1)	51 (6.5)		
Education				19.10	<.001
Elementary school	131 (13.4)	41 (21.7)	90 (11.5)		
Middle school	342 (35.1)	73 (38.6)	269 (34.2)		
High school	292 (29.9)	43 (22.8)	249 (31.7)		
College degree or above	210 (21.5)	32 (16.9)	178 (22.6)		
Marital status				7.28	.025
Married	901 (92.4)	182 (96.3)	719 (91.5)		
Other	74 (7.6)	7 (3.7)	67 (8.5)		
Occupation*				1.44	.486
Type I	488 (50.1)	100 (52.9)	388 (49.4)		
Type II	222 (22.8)	37 (19.6)	185 (23.5)		
Type III	265 (27.2)	52 (27.5)	213 (27.1)		
Domicile				2.01	.156
Local	157 (16.1)	24 (12.7)	133 (16.9)		
Non-local	818 (83.9)	165 (87.3)	653 (83.1)		
Smoking				20.16	<.001
Yes	185 (19.0)	54 (28.6)	131 (16.7)		
Formerly	49 (5.0)	15 (7.9)	34 (4.3)		
No	741 (76.0)	120 (63.5)	621 (79.0)		
Alcohol drinking				4.07	.044
Yes	386 (39.6)	87 (46.0)	299 (38.0)		
No	589 (60.4)	102 (54.0)	487 (62.0)		
Physical activity				0.38	.826
Low	208 (21.3)	42 (22.2)	166 (21.1)		
Middle	401 (41.1)	74 (39.2)	327 (41.6)		
High	366 (37.5)	73 (38.6)	293 (37.3)		
Sedentary time				3.29	.348
<2 h	84 (8.6)	21 (25.0)	63 (75.0)		
2–3.9 h	245 (25.1)	40 (16.3)	205 (83.7)		
4–5.9 h	223 (22.9)	43 (19.3)	180 (80.7)		
≥6 h	432 (43.4)	85 (20.1)	338 (79.9)		

MS = metabolic syndrome.

* Type I includes agricultural, forestry, animal husbandry, fishery and water conservancy production personnel, transportation equipment operators, commercial and service personnel and other workers. Type II includes personnel from government agencies, party and mass organizations, enterprises, public institutions, professional and technical personnel, military personnel and students. Type III includes unemployed, household workers and retired personnel.

### 3.2. Dietary pattern classifications

We subjected the intake of various food categories to factor analysis. The results showed a Kaiser–Meyer–Olkin statistic of 0.73, and Bartlett sphericity test resulted in a significance level (*P* < .001), indicating that the dietary intake data for this population were suitable for factor analysis. After applying maximum orthogonal rotation, 3 common factors with eigenvalues greater than 1 were identified. These factors explained 14.99%, 16.20%, and 18.03% of the dietary variation, contributing to a total variance of 49.22%.

The results of the factor analysis revealed 3 main dietary patterns in our study: (1) traditional pattern, which is similar to the traditional Chinese dietary pattern, with a focus on grains, vegetables, and animal meat; a total of 366 individuals (37.5%) were classified into this pattern. (2) Coastal pattern: this pattern was characterized by a higher intake of sea fish and seafood, dairy and dairy products, and fruits, including 283 individuals (29.0%). (3) Meat pattern: this pattern was centered around freshwater fish, poultry, and eggs, with 326 individuals (33.4%) falling into this category, as shown in Table 2.

**Table 2:** Food factor load of dietary patterns.

Category	Traditional pattern	Coastal pattern	Meat pattern
Grain	**0.79**	0.02	‐0.08
Vegetable	**0.52**	0.20	0.22
Fruit	0.13	**0.54**	0.33
Poultry	0.28	0.14	**0.58**
Livestock meat	**0.54**	0.02	0.35
Freshwater fish	‐0.05	0.23	**0.71**
Sea fish and seafood	‐0.04	**0.70**	0.23
Egg	0.16	‐0.05	**0.64**
Milk and milk products	0.18	**0.75**	‐0.21

The bold values indicate the main components of the dietary pattern identified under the reviewer's suggestion.

### 3.3. Prevalence of MS and its components in different dietary patterns

Each individual was assigned a score for the 3 dietary patterns, which were classified based on the maximum score. The prevalence of MS and its components in different dietary patterns is presented in Table 3. The results indicated statistically significant differences in the prevalence of MS (χ²=15.06, *P* < .001), elevated blood glucose (χ²=7.81, *P* = .020), elevated blood pressure (χ²=22.72, *P* < .001), and dyslipidemia (χ² = 7.02, *P* = .030) among the different dietary patterns. However, no statistically significant differences in the prevalence of central obesity and low HDL-C levels were found across the different dietary patterns.

**Table 3:** Comparison of the prevalence of MS and its components under different dietary patterns.

	MS	Abdominal obesity	High blood glucose	Elevated blood pressure	High TG	Low HDL-C
Traditional pattern	94 (49.7)	123 (42.0)	46 (51.1)	140 (46.2)	119 (41.8)	120 (40.8)
Coastal pattern	40 (21.2)	72 (24.6)	20 (22.2)	59 (19.5)	66 (23.2)	82 (27.9)
Meat pattern	55 (29.1)	98 (33.4)	24 (26.7)	104 (34.3)	100 (35.1)	92 (31.3)
χ^2^	15.60	5.06	7.81	22.72	7.01	1.97
*P*	<.001*	.080	.020	<.001†	.030‡	.373

HDL-C = high-density lipoprotein cholesterol, MS = metabolic syndrome, TG = triglyceride.

* Incidence of MS in the traditional pattern is higher than that in the coastal and carnivorous pattern.

† Prevalence of elevated blood pressure in the traditional and meat pattern is higher than that in the coastal pattern.

‡ Prevalence of high TG in the traditional pattern is higher than that in the coastal pattern.

### 3.4. Associations between dietary patterns and MS and its components

In Model 1, adjustments were made for 5 sociodemographic characteristics: gender, age, educational level, marital status, and occupation. The traditional pattern was used as the reference group because it had the largest proportion in the study. Multiple-factor logistic regression analysis revealed no significant association between MS and its components or dietary patterns. Model 2 was further adjusted for smoking, alcohol consumption, physical activity and sedentary time, based on Model 1. The results indicated no significant association between MS and its components or dietary patterns, as shown in Table 4.

**Table 4:** Logistic regression analysis of dietary patterns with MS and its components.

	MS	Abdominal obesity	High blood glucose	Elevated blood pressure	High TG	Low HDL-C
	OR (95% CI)	OR (95% CI)	OR (95% CI)	OR (95% CI)	OR (95% CI)	OR (95% CI)
Model 1						
Traditional pattern	1.00	1.00	1.00	1.00	1.00	1.00
Coastal pattern	0.67 (0.45, 1.00)*	0.93 (0.67, 1.31)	0.59 (0.34, 1.02)	0.93 (0.65, 1.32)	1.06 (0.5, 1.49)	0.89 (0.63, 1.26)
Meat pattern	0.75 (0.48, 1.17)	0.86 (0.59, 1.25)	0.78 (0.43, 1.41)	0.71 (0.48, 1.07)	0.92 (0.63, 1.35)	1.10 (0.75, 1.59)
Model 2						
Traditional pattern	1.00	1.00	1.00	1.00	1.00	1.00
Coastal pattern	0.67 (0.45, 1.00)†	0.95 (0.68, 1.34)	0.59 (0.34, 1.03)	0.91 (0.64, 1.30)	1.05 (0.74, 1.48)	0.88 (0.62, 1.25)
Meat pattern	0.79 (0.50, 1.24)	0.90 (0.62, 1.31)	0.79 (0.44, 1.45)	0.70 (0.47, 1.05)	0.94 (0.64, 1.39)	1.14 (0.78, 1.66)

HDL-C = high-density lipoprotein cholesterol, MS = metabolic syndrome, TG = triglyceride.

* *P* = 0.05.

† *P* = 0.051.

## 4. Discussion

In the culturally diverse coastal region of Pingshan District in Shenzhen, a rapidly urbanized area in southern China, our study aimed to discern and delineate the predominant dietary patterns among adult residents, ultimately identifying 3 primary patterns: the traditional, coastal, and meat patterns. The main finding of our study was that in the univariate analysis, the coastal pattern exhibited notably lower prevalence rates of MS, elevated blood pressure, and elevated triglyceride levels. However, upon further investigation using multivariate analysis, the study yielded results devoid of significant associations between dietary patterns and MS or its components. Notably, the analysis generated a *P*-value that approached the .05 significance threshold for the relationship between MS and dietary patterns, underscoring the need for additional validation through larger-sample studies to corroborate this association.

In this study, the MS prevalence of 19.4% was lower than the rates reported in other Chinese studies. The Chinese Nutrition and Health Survey data from 2015 to 2017 reported a standardized prevalence of MS of 31.1% among Chinese adults.^[4]^ Another study in 2017 indicated a 25.59% prevalence of MS among adult residents of Beijing.^[14]^ In addition, nutrition and dietary surveys conducted in Jiangsu Province in 2002, 2007, and 2014 found that 28.5% of study participants had MS.^[10]^ The lower prevalence of MS in our study may be associated with the unique urban characteristics of Shenzhen City. Being emblematic of China’s reform and opening-up, and with a substantial immigrant population, Shenzhen hosts an influx of migrant workers and a concentration of younger residents. Relatively high physical activity levels may contribute to maintaining overall health and subsequently reducing the risk of MS development given that younger populations exhibit lower MS prevalence rates.^[15,16]^ These findings highlight the impact of cultural diversity and urbanization in regions such as Shenzhen, underlining the need for tailored public health strategies adapted to the unique features of such areas. Our study provides valuable insights for addressing MS in rapidly urbanizing regions undergoing demographic shifts. Subsequent studies should explore the multifaceted aspects of MS in the context of evolving urban centers.

The dietary patterns we identified, including the traditional, meat, and coastal patterns, exhibiting congruent with prior research findings. A study examining dietary pattern trends in the Chinese population^[17]^ similarly recognized traditional meat patterns. The traditional dietary pattern is distinguished by its high content of dietary fiber, vitamins, minerals, and diverse nutrients, coupled with low saturated fat levels. The merits of this dietary pattern include a reduced risk of oxidative damage, metabolic regulation, and the moderation of blood lipid levels, thereby exerting influence on the occurrence and development of MS.^[18]^ In contrast, meat patterns are more prevalent in economically developed western countries. Rapid economic and social progress in China and the elevated living standards of its residents have driven a gradual increase in the consumption of animal-based foods^,[19]^ fostering a predominantly animal-based dietary pattern in China. The meat pattern is marked by higher fat content, increased energy density, and substantial levels of saturated fats and cholesterol. Prolonged adherence to this dietary pattern can escalate the risk of obesity and cardiovascular diseases,^[20]^ which, in turn, may heighten susceptibility to developing MS. Existing research findings support this perspective.^[21]^

Our study identified a coastal pattern, characterized by elevated seafood consumption, which is congruent with results from dietary pattern investigations^[22]^ conducted in other coastal regions. Furthermore, in the inland regions of China, such as Guizhou and Sichuan,^[11,23]^ dietary patterns linked to seafood consumption have surfaced. This trend could be attributed to the rapid advancements in modern logistics and cold chain technologies, facilitating the rapid transportation of fresh seafood products from their sources to various parts of the country, significantly enhancing seafood accessibility. The coastal pattern is abundant in high-quality proteins, vitamins, minerals, and unsaturated fatty acids, particularly Omega-3 fatty acids. These constituents are associated with functions^[24,25]^ related to lipid metabolism regulation, improved insulin sensitivity, and modulation of gut microbiota metabolism, all of which may positively affect MS prevention.

The existing body of literature exhibits mixed findings regarding the relationship between dietary patterns and MS. While some studies have validated this association,^[23,26]^ others have failed to identify a significant link between dietary patterns and MS. Instead, they have observed associations between dietary patterns and specific components of MS.^[11]^ In the current investigation, we did not identify a significant association between dietary patterns and MS or its individual components. Multiple factors could contribute to these findings, including a relatively modest sample size, a limited spectrum of surveyed foods, the potential presence of recall bias (as food frequency surveys rely on participants’ recollection of their food consumption over the past year), participants’ imprecise estimation of food portion sizes, and absence of data concerning cooking methods for the foods considered in our study. Furthermore, numerous studies have already indicated a close association between ultra-processed foods and metabolic syndrome. However, the current study did not investigate ultra-processed foods, which is also one of its limitations.

Our study had several strengths. Initially, it was conducted within the Pingshan District in Shenzhen, a nationally designated demonstration area for comprehensive chronic disease prevention and control. Within this district, specialized infrastructure for chronic disease prevention and control has been established, encompassing disease control centers, healthcare facilities, and community health centers. This framework provides an optimal environment for research on chronic diseases. Moreover, the study was conducted under the guidance of the Shenzhen Municipal Health and Family Planning Commission and methodically executed by the Shenzhen Chronic Disease Control Centre. This oversight ensured a uniform and standardized survey process, guaranteeing the reliability of the data collected. Furthermore, our research was characterized by a meticulously designed survey questionnaire that captured not only dietary habits but also a broad spectrum of health-related behaviors. This comprehensive approach enabled a thorough evaluation of the participants’ lifestyles, rendering the study a valuable reference for gaining insight into the connection between dietary patterns and health outcomes.

In conclusion, our study did not reveal a significant correlation between dietary patterns and MS, after adjusting for relevant covariates. However, our findings underscore the potential health advantages associated with coastal dietary patterns, particularly in light of the presence of unsaturated fatty acids, such as Omega-3 fatty acids derived from seafood. These fatty acids play a distinctive role in the regulation of metabolism and mitigation of cardiovascular disease risk, with limited substitution options from alternative food sources. As a result, pertinent health authorities should be involved in chronic disease and cardiovascular disease prevention and control to consider elevating the prominence of coastal dietary patterns. In the dissemination of nutritional knowledge and relevant information, emphasis should be placed on elucidating the advantageous impact of nutrients derived from seafood on overall health.

## Acknowledgments

We would like to thank all the authors for their great efforts in this research.

## Author contributions

**Conceptualization:** Maozhen Fu, Dandan Yang.

**Formal analysis:** Dandan Yang.

**Funding acquisition:** Maozhen Fu.

**Investigation:** Maozhen Fu, Yan Luo.

**Methodology:** Dandan Yang.

**Project administration:** Maozhen Fu.

**Software:** Maozhen Fu.

**Supervision:** Yan Luo.

**Validation:** Yan Luo.

**Visualization:** Yuliang Zou.

**Writing – original draft:** Maozhen Fu, Dandan Yang, Yuliang Zou.

**Writing – review & editing:** Maozhen Fu, Dandan Yang, Yan Luo, Yuliang Zou.
